# Editorial: Augmenting human experience and performance through interaction technologies

**DOI:** 10.3389/fpsyg.2024.1356658

**Published:** 2024-03-07

**Authors:** Giacinto Barresi, Hasan Ayaz, Jean-Marc Seigneur, Giovanni Di Pino, Marta Bertolaso

**Affiliations:** ^1^Rehab Technologies Lab, Istituto Italiano di Tecnologia, Genoa, Italy; ^2^School of Biomedical Engineering, Science and Health Systems, Drexel University, Philadelphia, PA, United States; ^3^Department of Psychological and Brain Sciences, College of Arts and Sciences, Drexel University, Philadelphia, PA, United States; ^4^Drexel Solutions Institute, Drexel University, Philadelphia, PA, United States; ^5^A. J. Drexel Autism Institute, Drexel University, Philadelphia, PA, United States; ^6^Department of Family and Community Health, University of Pennsylvania, Philadelphia, PA, United States; ^7^Center for Injury Research and Prevention, Children's Hospital of Philadelphia, Philadelphia, PA, United States; ^8^CUI Medi@LAB, University of Geneva, Genéve, Switzerland; ^9^NEXT: Neurophysiology and Neuroengineering of Human-Technology Interaction Research Unit, Universitá Campus Bio-Medico di Roma, Rome, Italy; ^10^Research Unit of Philosophy of Science and Human Development, Universitá Campus Bio-Medico di Roma, Rome, Italy

**Keywords:** human augmentation, augmented human, augmented reality, augmented cognition, human-robot interaction, wearable robotics, cyborg

The challenge of enhancing human performance and capabilities spanning sensory, cognitive, and motor functions has become a focal point of interest within converging interdisciplinary research fields. These fields, ranging from neuroengineering and neuroergonomics to human factors and rehabilitation, reflect a growing commitment to understanding and advancing human potential (Clark and Parasuraman, 2014; Ayaz and Dehais, 2019; Valeriani et al., 2021). The pursuit of enhancement seeks to transcend the limits of human experience and performance. This could be achieved by three cardinal research directions: (i) exploring human neurobiology and brain function as the foundational substrate for cognition and information processing, (ii) understanding the intricate machinery or computer systems that humans engage with for a task, and (iii) examining the interface serving as the interaction medium, facilitating bidirectional information exchange between humans, and the said systems.

In the context of our Research Topic, we directed our attention specifically to the third aspect augmenting human capabilities through interaction technologies such as Virtual Reality (VR), Augmented Reality (AR), or Mixed Reality (MR). These cutting-edge technologies form the nexus of our exploration, acting as transformative tools in enhancing human potential through immersive and dynamic interfaces.

The capability of interaction technologies to enhance the human skills leads to the augmentation of our the individual experience and performance (Raisamo et al., 2019). We may consider, for instance, the different ways to merge virtual and real items in the same context, beyond the VR conditions of a fully digital environment. AR (based on the overlay of digital items on a physical setting) and MR (based on the integration of digital items in a physical setting, making the first behaving like objects of the second) show an impressive potential in improving our abilities. There is currently a debate on the definitions of these forms of extended reality (XR - however, originally, mixed reality was adopted as the set of all combinations of virtual and real items) (Milgram et al., 1995; Papadopoulos et al., 2021; Skarbez et al., 2021; Rauschnabel et al., 2022). Such a discussion may clearly foster interdisciplinary studies for pondering the emergence of novel interaction patterns, especially when other challenging systems—as in the case of Artificial Intelligence (AI) to integrate and assist the human intelligence (Chignell et al., 2023)—may increase the complexity of our context.

Accordingly, the collection of papers presented in this Research Topic includes examples of perspectives and studies on the augmentation of human experience and performance from different points of view according to their field of application.

First of all, through a paper titled *Degree of enhancement: A theoretical and formal definition*, Cassioli and Balconi face the challenge of a formal definition of enhancement. The authors propose the concept of Degree of Enhancement (DoE) to embrace the complexity of different forms of augmentation in complex socio-technical systems with the human factors and values. DoE can certainly become a powerful tool for designer of human-centric augmented systems.

This Research Topic also considered systems design in terms of aspects affecting perception and user experience. For instance, Ashtiani et al. presents their work on the *Impact of motion cues, color, and luminance on depth perception in optical see-through AR displays* to discuss the factors listed into the title according to experimental studies that can offer novel guidelines to AR developers. Furthermore, Sinlapanuntakul et al. propose their manuscript about *Exploring the user experience (UX) of a multi-window augmented reality environment* to investigate user-centered design studies encompassing usability and mental workload too. Their work offers impactful (qualitative and quantitative) insights in design, especially about hand-tracking solutions for AR.

After presenting these studies on general-purpose design of interactions, it can be noticed how the papers in this Research Topic explore two potential areas of application of AR and MR systems: the first one involves younger and older users in urban contexts: the second targets medical domains to assist their users.

About the first set, the approach described by Reaver in *Augmented reality as a participation tool for youth in urban planning processes: Case study in Oslo, Norway* explores the use of AR in a participatory urban planning case, the one of the Oslo Trees project in Norway, involving young people in both design and learning activities. On the other hand, Van et al. set their research on the *Evaluation of assistance systems allowing older drivers to intercept moving inter-vehicular space*. The authors performed tests through a simulator, observing the opportunity of designing elderly-centered Advanced Driving Assistance Systems (ADAS).

The second set includes both one case on MR in surgery and one about the co-design of augmentative artificial intelligence (AI). About the case on MR, Fick et al. propose their works about *Comparing the influence of mixed reality, a 3D viewer, and MRI on the spatial understanding of brain tumours*. Through a task requiring to align a virtual tumor with the patient's anatomy, the authors observed the high precision derived from using MR in surgical pre-operative planning.

Finally, the work of Ventura et al. about *Co-designing an interactive artificial intelligent system with post-stroke patients and caregivers to augment the lost abilities and improve their quality of life: a human-centric approach* allows to highlight how the topic of human augmentation can be extended beyond AR and MR. Indeed, the authors discussed the potential of an AI system, MAIA, to interpret the intentions of assistive device users. Their scope is to assist post-stroke patients and caregivers in restoring the motor autonomy of the first. However, they also observe the need for reaching higher user trust in exploiting such advances.

Summing up, this Research Topic surely offered a useful opportunity for organizing studies on the augmentation of human experience and performance. This may constitute an example for preparing additional initiatives devised to understand the role of augmentation in human-machine systems and to develop more and more sustainable and integrated technologies designed for helping people.

## Author contributions

GB: Writing—original draft, Writing—review & editing. HA: Writing—original draft, Writing—review & editing. J-MS: Writing—original draft, Writing—review & editing. GD: Writing—original draft, Writing—review & editing. MB: Writing—original draft, Writing—review & editing.
